# The kinetic Orbison illusion

**DOI:** 10.1177/20416695231196979

**Published:** 2023-09-06

**Authors:** Hikari Matsunaga, Hiroyuki Ito, Tama Kanematsu

**Affiliations:** Graduate School of Design, 12923 Kyushu University, Fukuoka, Japan; Faculty of Design, 12923 Kyushu University, Fukuoka, Japan; Center for Applied Perceptual Science, 12923 Kyushu University, Fukuoka, Japan

**Keywords:** geometrical illusion, Orbison illusion, motion streaks, induced motion, aperture problem

## Abstract

In the typical Orbison illusion, the sides of a square placed on concentric circles appear to be bending toward the center of the circles. We report a motion version of the Orbison illusion (namely, the kinetic Orbison illusion). When a dot moves along a square trajectory against a background of concentric circles, the sides of the trajectory appear to bend toward the center and the corners appear to be sharpened. In the present study, observers adjusted the shape of a comparison stimulus to the shape of the perceived trajectory by bending the sides. The amount of illusion was operationally defined as the largest discrepancy between the square and adjusted shape in the comparison stimulus. It was found that the illusory bending was more than twice the static Orbison illusion and reached a maximum of 7.3% of the length of one side. Experiments including a comparison between fixation and pursuit conditions revealed that the main cause of the kinetic illusion was not motion streaks of the dot crossing background circles. We propose an alternative hypothesis based on induced motion generated by background circle motion, the direction of which is misperceived owing to the aperture problem.

The perceived trajectory of a moving object is sometimes altered by the background, resulting in an illusion of motion direction (e.g., Anstis, 2012; Cesàro & Agostini, 1998; Ito & Yang, 2013; Khuu, 2012; Swanston, 1984). In this article, we add a clear example of this type of phenomenon to the literature. The trajectory of a dot moving in a square path on concentric circles is perceived to be bent inward, which we call the kinetic Orbison illusion (see Figure 1). The Orbison illusion is one of the geometric illusions reported by Orbison (1939), in which a square is placed on concentric circles, and the sides of the square are perceived to be bent inward. The kinetic Orbison illusion can be considered as an illusion that replaces the straight line of the Orbison illusion with a motion trajectory of a dot.

**Figure 1.**
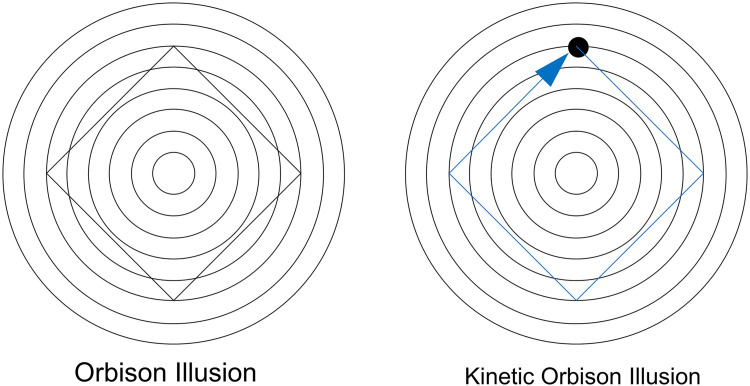
Kinetic Orbison illusion. The perceived motion trajectory of a dot is perceived to bend inward (right panel) although the physical motion path is a square as in the traditional Orbison illusion figure (left panel). Please try Supplemental Movie 1 for a demonstration of the effect.

Although no previous study on the kinetic Orbison illusion has been reported, a few studies have reported kinetic versions of geometrical illusions (Khuu, 2012; Swanston, 1984). Swanston (1984) reported that when the parallel lines of the Hering illusion figure (Hering, 1861) or long lines of the Zöllner illusion figure (Zöllner, 1860) were replaced by a motion path of a dot, the same illusion tendency in the motion direction or trajectory as that of the stationary figures was observed. Swanston (1984) believed that subjective contours generated when observing the moving dot produced the illusion and that the illusory motion trajectories were common to the illusion in the stationary figures; that is, the acute angle was overestimated at each intersection between the oblique lines and the subjective contours as well as actual contours. The largest effect was acquired at a relative angle of 10°. Later, Cesàro and Agostini (1998) showed the illusory effect of intersections between short lines and a straight motion path of a dot on the perceived motion trajectory. They reported that in the *slalom effect*, a dot moving on a straight path crossing tilted short line segments forming a zigzag pattern was seen to move on a sinusoidal path. The illusion was inversely proportional to the size of the incidence angle and velocity. Gheorghes et al. (2020) confirmed a perceptual bias toward perpendicularity between the dot motion trajectory and short lines as a possible cause of the *slalom effect*.

The kinetic Zöllner illusion was also studied by Khuu (2012). He investigated the relationship between the strength or salience of motion streaks of dots and the amount of illusion in the kinetic Zöllner illusion, varying the speed, contrast, and lifetime of dots. As the amount of illusion decreased or disappeared under conditions that shortened or weakened motion streaks (i.e., a low speed, low contrast, and short lifetime of the dots), he concluded that the kinetic Zöllner illusion is caused by mutual suppression between orientation-selective cells responding to motion streaks and orientation-selective cells responding to background lines when the two orientations are close to each other (e.g., 15° in his study). Contrarily, Gheorghes et al. (2020) tested the necessity of pursuit eye movement for the *slalom effect* and found that the effect arose during observation with fixation that caused motion streaks as well as observation with pursuit eye movement that weakened motion streaks. Swanston (1984) and Cesàro and Agostini (1998) did not describe the observing condition (i.e., whether observers fixated on a point or viewed the figure freely), preventing discussion of the effect of motion streaks. However, the studies noted above may be common in assuming a pseudo-static geometrical illusion arising after motion information was translated into orientation information of the motion path.

In fact, it has been revealed that the determination of the motion direction has a close relationship with orientation-selective cells. Burr (1981) showed a temporal summation of a rapidly moving object to ∼ 100 ms, suggesting a possible translation of the motion trajectory into orientation information through neural blur or streaks (Burr & Morgan, 1997). Geisler (1999) proposed a model of a motion detection mechanism, combining motion-direction-selective cells responding to motion in a perpendicular direction of an edge orientation and orientation-selective cells responding to motion streaks. Actually, there is an overlap between cells responding to a static image implying motion (glass pattern) and those responding to real motion (Krekelberg et al., 2005). Apthorp and Alais (2009) showed that motion streaks induce tilt aftereffects and tilt illusions, indicating mutual inhibition in orientation detection between motion streaks and real gratings. Although this combination of direction-selective cells and orientation-selective cells provides precise motion direction information (Geisler, 1999), when the orientation-selective cells responding to motion streaks interact with those responding to real edges as in the work of Apthorp and Alais (2009), the signaled motion direction may be affected (Khuu, 2012). Thus, according to this theory, the motion processing mechanism for precise determination of the motion direction may work in reverse and result in the illusion of the motion direction when the motion direction is close to the background line orientation.

The present article qualitatively and quantitatively describes a new phenomenon, the kinetic Orbison illusion, and examines the hypothetical mechanism assuming motion streaks. In Experiment 1, we quantitatively examined whether the same illusion is observed in the kinetic Orbison illusion as in the Orbison illusion with stationary figures. In Experiment 2, we examined the effect of the square motion-path orientation. In Experiment 3, we examined the effect of the number of circles and the effect of motion streaks by comparing a fixed viewpoint condition and a smooth-pursuit condition where observers tracked the moving dot with their eyes, as tested by Gheorghes et al. (2020). In Experiment 4, we examined the effects of the inner and outer circles. In Experiment 5, we examined the effect of the dot speed. Finally, we propose an alternative theory that does not depend on motion streaks.

## Experiment 1

Experiments 1 and 2 were conducted to confirm the phenomenon of the kinetic Orbison illusion, to quantitatively demonstrate the illusion, and to compare the illusion with the static Orbison illusion.

### Method

#### Participants

There were 10 participants (age: mean of 24.9 years, standard deviation [SD] of 3.53 years, nine females and one male). The visual acuity of the participants was normal or corrected to normal. Written informed consent was obtained from each participant.

In all the reported experiments, we determined the number of participants referring to the past research cited in the “Introduction” section. In addition, to conduct Mauchly tests for sphericity in the repeated measures analysis of variance (ANOVA), the number of participants was determined to be equal to or more than the number of conditions in Experiments 3, 4, and 5.

#### Apparatus

Visual stimuli were produced on a personal computer (DELL, D03M005) and displayed on an organic light-emitting diode display (SONY, PVM-A250). The display had a refresh rate of 60 Hz and dimensions of 31 cm (vertical) by 55 cm (horizontal) (31° × 55° in visual angle). A chinrest was used to maintain a constant viewing distance of 57.3 cm.

#### Stimuli

**Figure 2.**
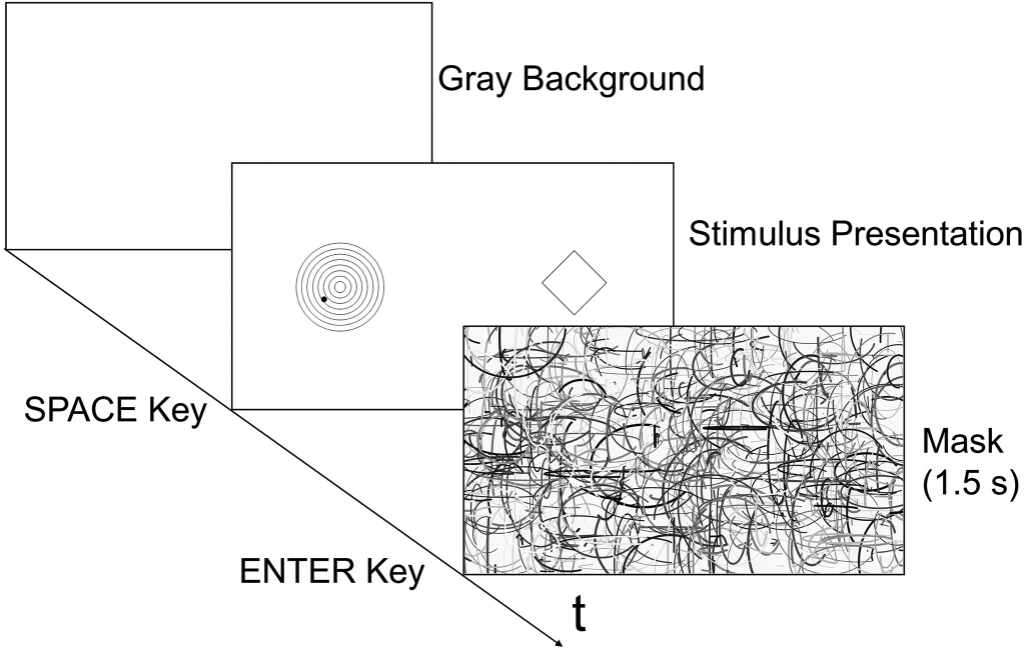
The sequence of a trial.

At the beginning of the trial, only a gray background was presented. The stimulus was presented when a participant pressed the SPACE key on a keyboard. The standard stimulus was presented on the left of the screen and the comparison stimulus was on the right (see Figure 2). The stimuli were presented until the participant pressed the ENTER key. The standard stimulus was the static Orbison illusion figure (see Figure 1, left panel) or the kinetic Orbison illusion movie (see Figure 1, right panel). The presented stimuli (concentric circles, a square, and a moving dot) were drawn in black (0.32 cd/m²) on a gray background (16.5 cd/m²).

The kinetic Orbison illusion movie consisted of a moving dot and eight concentric circles. The width of a line was 0.0295°. The outermost circle had a diameter of 10.0° and the innermost circle had a diameter of 1.3°. The diameter of the moving dot was 0.28°, and the motion path was a square with a side of 5.9°. The dot moved clockwise at 4.43°/s (5.3 s/rev). The position of the dot was refreshed at a rate of 30 times per second.

**Figure 3.**
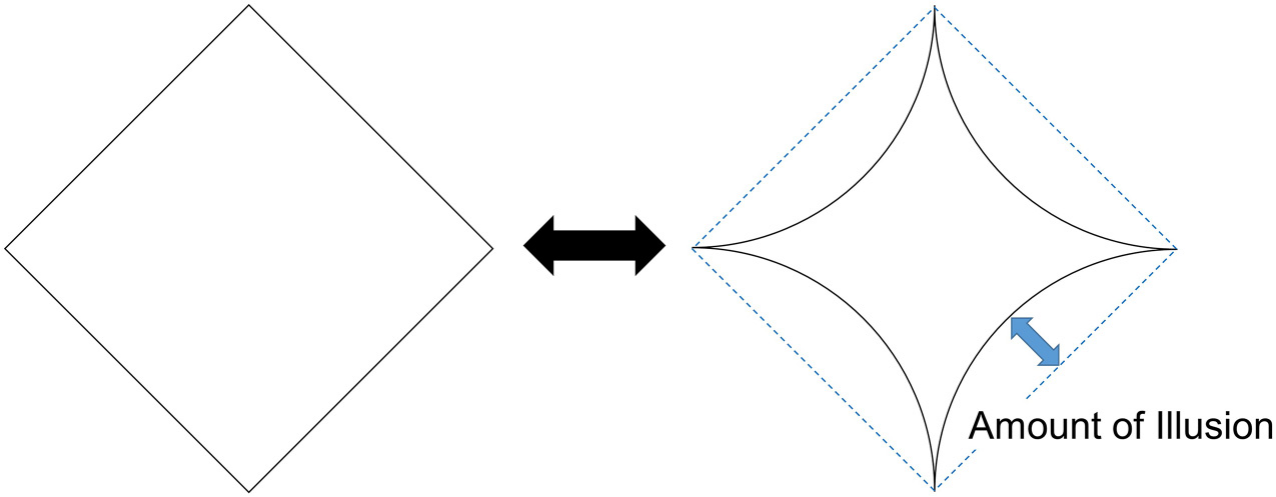
Comparison stimulus and adjustment method. Participants used keys to control the curvature of the comparison stimulus. See details in the text.

The square motion path of the dot under the kinetic Orbison illusion condition and the square under the Orbison illusion condition had the same shape and size. The comparison stimulus was initially a square with a side of 5.9°, and each participant adjusted the straight line segments to curved line segments to produce a star-shaped figure (see Figure 3). The degree of curvature was varied by changing the radii of curvature of the circles passing through the ends of the line segment. The comparison stimulus was similar in size to the motion path of the dot or the square in the standard stimulus.

When the participant finished the adjustment and pressed the ENTER key, the mask stimulus was presented. On average, 24 arcs of random size and luminance were drawn at random positions on the screen 30 times per second as a mask (see Figure 2). This continued for 1.5 s.

#### Procedure

The experiment was conducted in a dark room. The stimuli were presented on the gray screen when a participant pressed the SPACE key. The participant's task was to freely observe the standard stimulus that was supposed to produce the Orbison illusion or the kinetic Orbison illusion and to adjust the comparison stimulus to the perceived shape of the static square or the motion trajectory of the dot. The amount of illusion was defined as the extent to which the line segment appeared concave inward from the square line segment of the comparison stimulus; that is, the distance from the center of the side segment to the center of the reproduced curve (see Figure 3). In the case that a reproduced line segment was straight, not concave at all, the illusion was considered to be zero. It is important to note that we did not assume that the illusion was produced only by the sides bending inward. It was possible to perceive a curved side or a curved trajectory of the dot motion from perceived acute corner angles. The matched comparison stimulus indicated the perceived *shape* of the square stimulus or the square trajectory of dot motion. Thus, the amount of illusion measured here is considered to be operationally defined.

Participants adjusted the amount of concavity of the line segment of the comparison stimulus with the up/down and left/right keys. The up/down and left/right keys were adjustable by ± 0.0295° and ± 0.1475°, respectively. Participants could repeat an observation and adjustment as many times as they wished. No fixation point was presented, and eye movement was not restricted. When participants pressed the ENTER key after completing the adjustment, the stimulus disappeared and the mask was presented.

There were a total of 16 trials per participant for two conditions (static Orbison illusion/kinetic Orbison illusion) and eight repetitions. All trials were performed in random order. Participants were allowed to take breaks at the preparation screen.

### Results and Discussion

**Figure 4.**
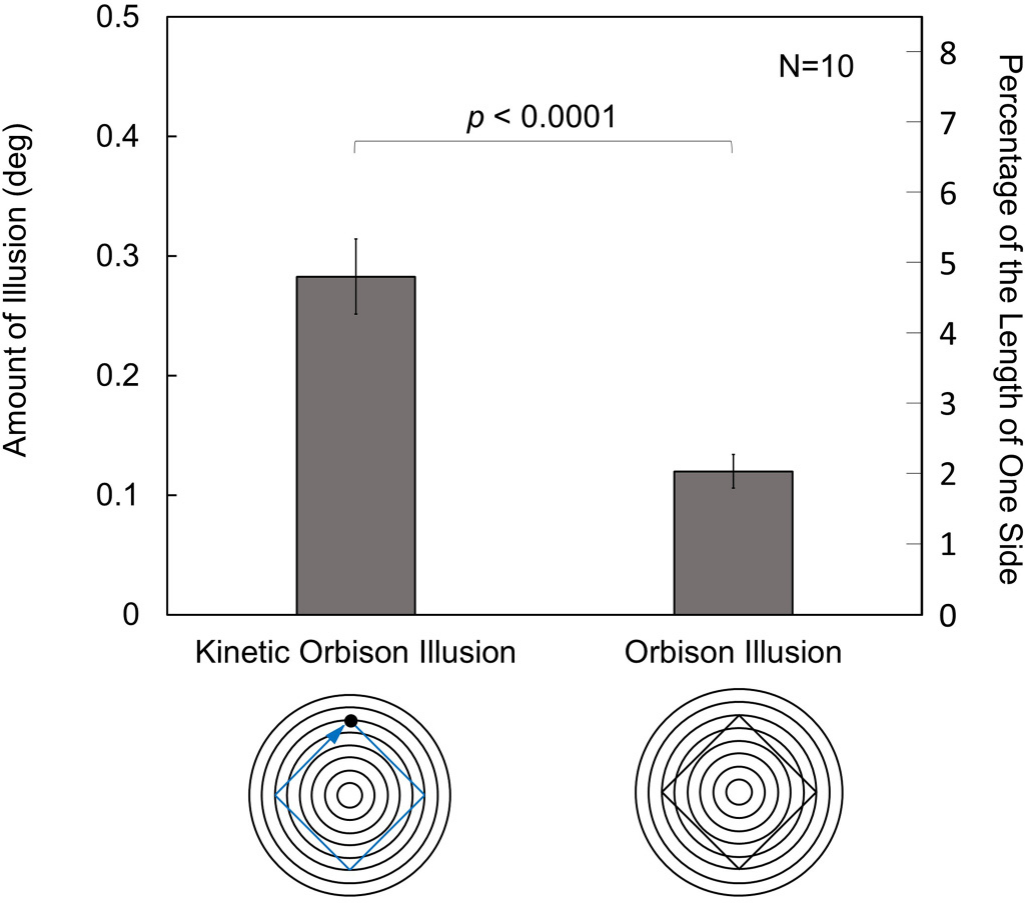
Results of Experiment 1. The kinetic Orbison illusion was larger than the static Orbison illusion. The error bars indicate standard errors of means (SEs). The *p* value indicates the result of a two-tailed paired *t*-test.

The results are shown in Figure 4. There was a significant difference in the amount of illusion between the kinetic Orbison illusion and static Orbison illusion, *t*(9) = 6.6, *p *< .0001, Cohen's *d* of 2.09. The results quantitatively demonstrate the existence of the kinetic Orbison illusion. Although the kinetic Orbison illusion is similar to the static Orbison illusion in which straight lines are perceived to be bent inward owing to the presence of concentric circles, the amount of the kinetic Orbison illusion was 2.36 times that of the static Orbison illusion. Here, the amount of the kinetic Orbison illusion was 4.79% of one side of the square path.

The measured amount of illusion indicates that the kinetic Orbison illusion was clearly observed. However, one could argue that the perceived distortion was simply caused by the nature of motion perception and not the interaction between the dot motion and the concentric circles because it is possible that the perceived distortion could be observed even without the circles. Another potential criticism relates to the orientation of the square. It is known that a square changes appearance into a diamond when it is tilted by 45° (i.e., the *square–diamond illusion*, Schumann (1900)). It is also known that the perception of oblique lines is less accurate than that of horizontal and vertical lines (i.e., the *oblique effect*, Appelle (1972)). Therefore, the diamond shape may have an illusory effect other than the size illusion. For example, the corner angle of a diamond may be perceived as a little less than 89° (Ito & Nose, 2021). This might result in the perceived bending of the sides even without background concentric circles. In the case of the motion trajectory, it is not clear whether the perceived path on a *diamond* is distorted by itself.

## Experiment 2

Experiment 2 was conducted to examine two factors that possibly affect the amount of kinetic Orbison illusion, namely the existence of the background concentric circles and the orientation of the square motion path. We attempted to separate the effect of the square orientation from the effect of the concentric circles.

### Method

#### Participants

There were 10 participants (age: mean of 25.6 years, SD of 5.87 years, seven females and three males).

#### Apparatus

The display was an organic light-emitting diode display (SONY PVM-2541).

#### Stimuli

**Figure 5.**
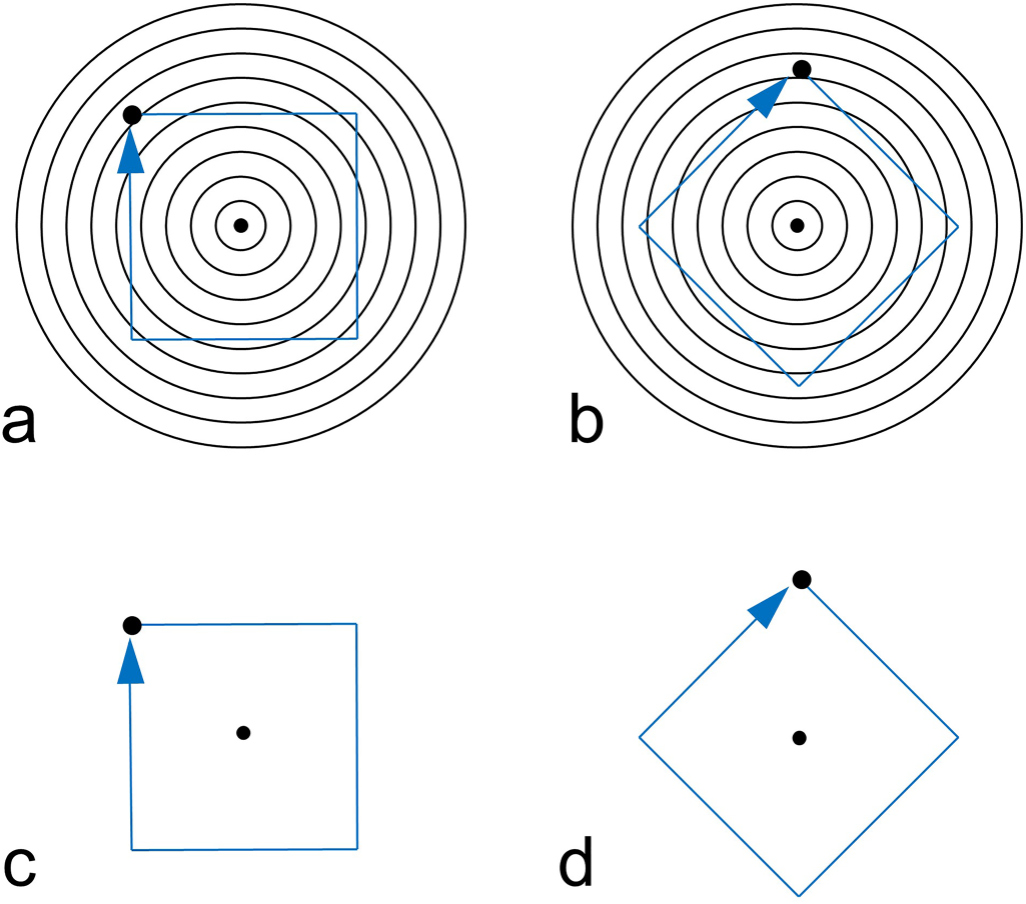
Schematic illustration of the stimuli used in Experiment 2. The upper panels show the kinetic Orbison illusion stimuli with an upright square path of the dot (a) and with a rotated path (b). Panels (c) and (d) show a stimulus with dot motion only, removing the circles in (a) and (b), respectively. The central dot in each panel indicates the fixation point.

The stimuli consisted of a moving dot and nine concentric circles. Under the condition that the concentric circles were presented, the outermost circle had a diameter of 10.0° and the innermost circle had a diameter of 1.1°. Under the condition that the concentric circles were not displayed, only a moving dot was presented. The orientation of the square motion path of the dot was upright at 0° or rotated through 45° (see Figure 5). The comparison stimulus was presented with the same orientation as the standard stimulus, at either 0° or 45°. There were thus four standard stimuli (i.e., combinations with/without concentric circles and 0°/45° orientation of the square path).

#### Procedure

The task of the participants was the same as that in Experiment 1. In Experiment 1, the participants viewed the standard stimuli freely. In Experiment 2, the priority was to draw a square with azimuths of 0° or 45° as the motion path of the dot on the retina, and a fixation dot was thus displayed at the center of the concentric circles. Participants were told to observe the motion of the dot while gazing at the fixation dot.

There were a total of 32 trials per participant for the two circle conditions (presence or absence of concentric circles), two orientation conditions (0° or 45° orientation of the square motion path), and eight repetitions. The trials were conducted in random order. Other procedures were the same as those in Experiment 1.

### Results and Discussion

**Figure 6.**
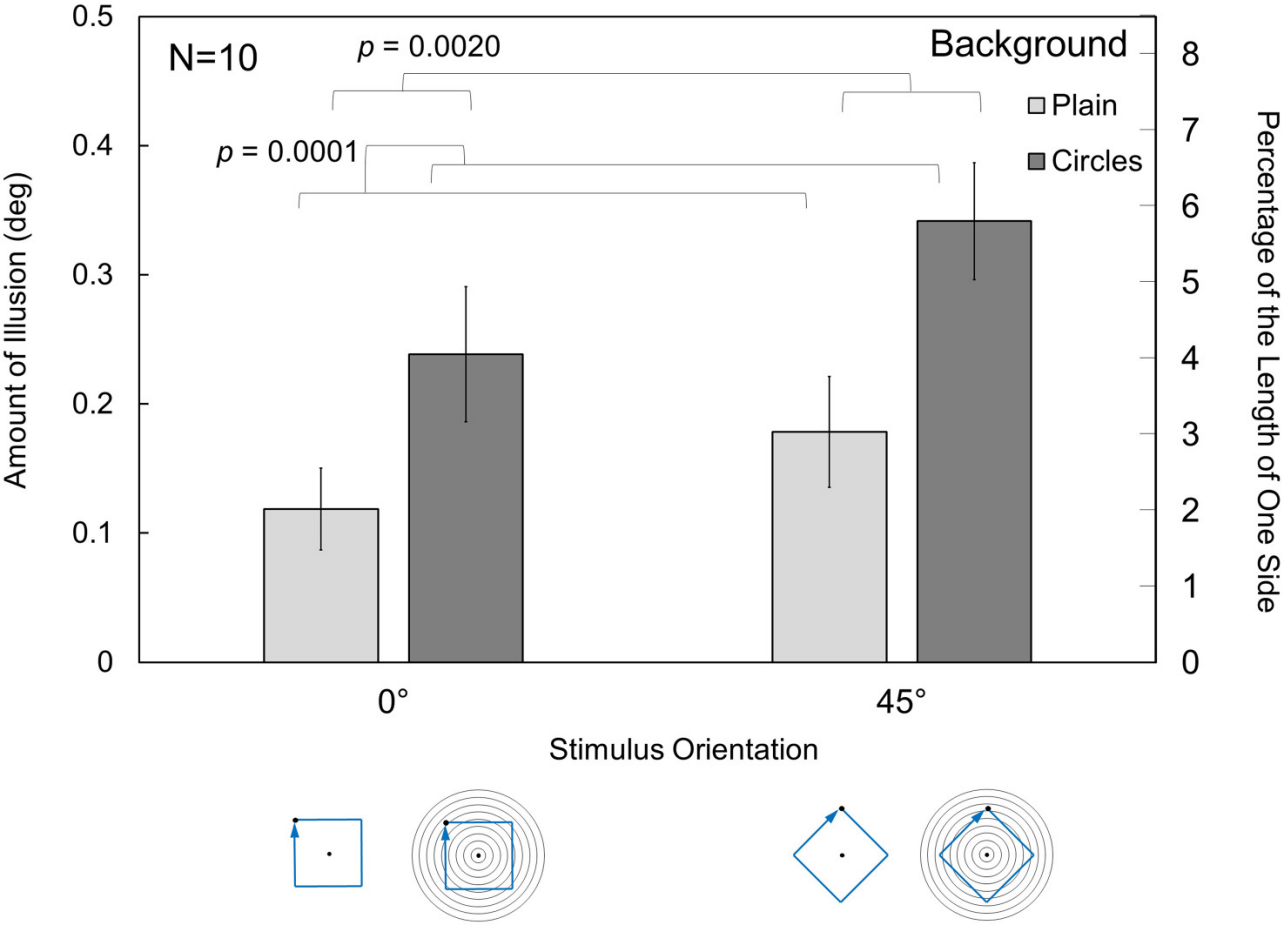
Results of Experiment 2. The effects of the orientation and background circles are clearly seen. The *p* values indicate significant differences in the main effects. The error bars indicate SEs. See details in the text.

The results of Experiment 2 are shown in Figure 6. A two-way repeated measures ANOVA revealed the main effects of the stimulus orientation, *F*(1,9) = 18.489, *p *= .0020, *η_p_*²* *= 0.673, and the presence of background circles, *F*(1,9) = 41.815, *p *= .0001, *η_p_*²* *= 0.823). The interaction was not significant, *F*(1,9) = 0.801, *p *= .3942, *η_p_*²* *= 0.082. The results show that the orientation of the square trajectory and the presence of the background circles are major determinants of the illusion.

The illusion was larger when the background circles were present than when they were absent. The concentric circles, therefore, affected the appearance of the linear trajectory of the dot, and we thus refer to this phenomenon as the kinetic Orbison illusion. However, it is also true that the illusion was observed even under the conditions without the background circles in this experiment. It is not clear whether this result is a different phenomenon caused by motion stimuli. We discuss this point in the “General Discussion” section.

The illusion was larger for a stimulus orientation of 45° than for an orientation of 0°, indicating that oblique motion produced a larger illusion than horizontal or vertical motion. We consider two reasons for the effect. The first is the same as that for the square–diamond illusion. As noted before, Ito and Nose (2021) showed that the corner angle of a diamond was perceived as < 89°. In the kinetic Orbison illusion, the corners drawn by the motion trajectories of a dot may also be perceived as having acute angles, enhancing the star-shaped appearance of the trajectory. The second is that the oblique orientation of the trajectories or the oblique motion direction may be perceived inaccurately, relative to the horizontal or vertical trajectories or those motion directions, as in the oblique effect for a static figure. The geometrical illusion in angle or direction (such as the Zöllner or Poggendorff illusion) is known to be stronger in the oblique orientation than in the vertical or horizontal orientation (Oyama, 1960). As motion trajectories may be more ambiguous in the perception of the orientation or corner angle than real lines, they may be more readily affected by illusion-inducing factors. The perception of the horizontal and vertical straight motion trajectories would be relatively robust against the illusory effect. We discuss the residual effect for the 0° condition without background concentric circles in the “General Discussion” section.

In summarizing the results of Experiment 2, we state that the kinetic Orbison illusion is effectively observed for the oblique orientation although there could be other factors affecting the illusion. In Experiment 3, we examined the effect of the number of concentric circles. Changing the number of concentric circles changed the number of intersections between the dot-motion path and the background circles. Experiment 3 further examined the effect of the observer's viewing condition (fixating or pursuing). In Experiment 2, as participants fixated their gaze on the central dot, the dot motion could produce motion streaks. We can test the effect of motion streaks on the kinetic Orbison illusion by controlling eye movement conditions. If motion streaks are important to this illusion, the illusion will weaken or be lost under the condition of pursuit eye movement because motion streaks of the dot will not be generated when the eyes pursue the moving dot.

## Experiment 3

In Experiment 3, we tested the effects of the number of concentric circles and eye movements on the kinetic Orbison illusion. The experiment was conducted for six conditions of the circle number (including no circles) and two viewing conditions (fixation and smooth pursuit).

### Method

#### Participants

There were 12 participants (age: mean of 25.08 years, SD of 4.13 years, nine females and three males).

#### Apparatus

The apparatus was the same as that in Experiment 1.

#### Stimuli

**Figure 7.**
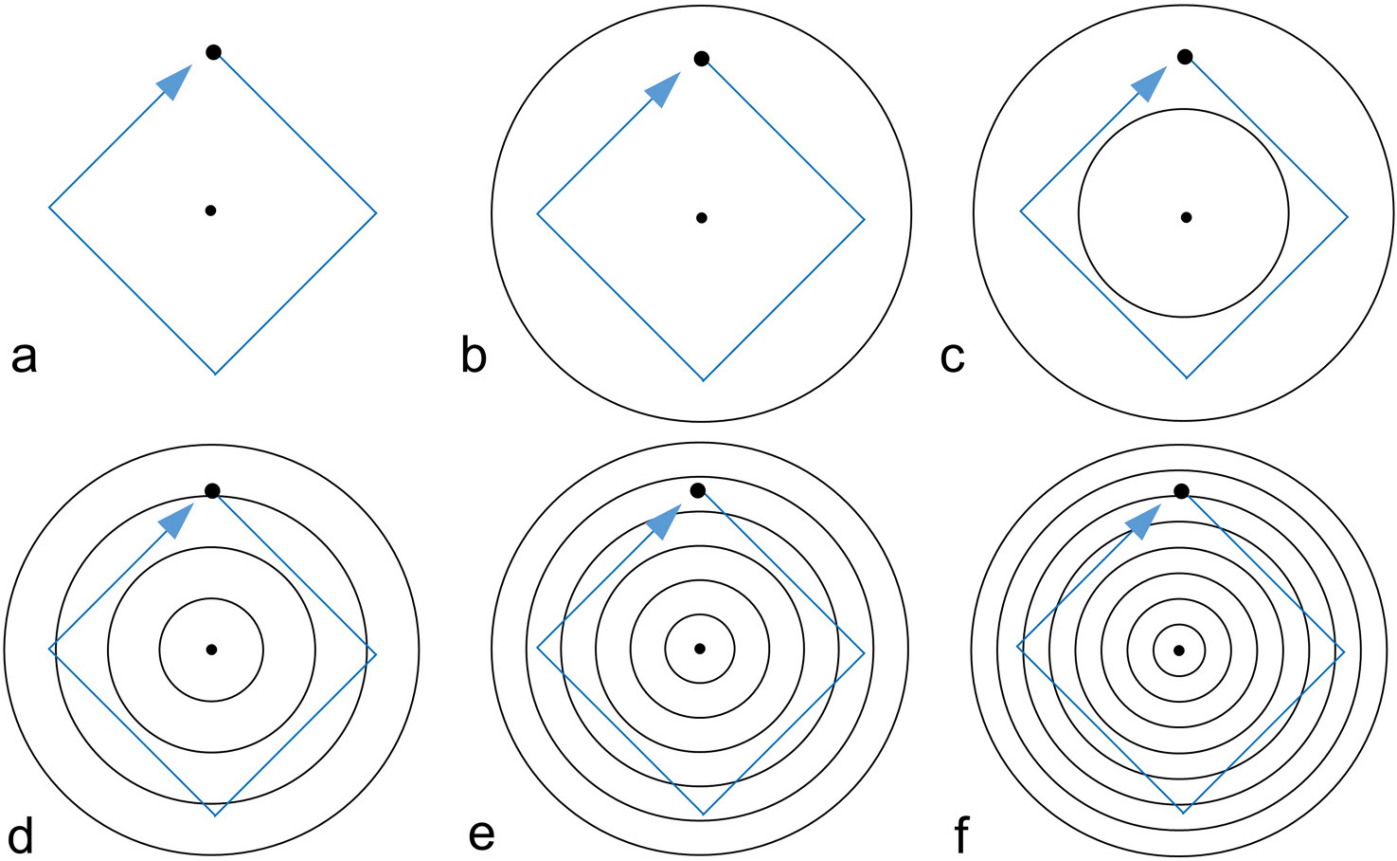
Schematic illustration of the stimuli used in Experiment 3. (a)–(f) The conditions of zero, one, two, four, six, and eight circles, respectively. The central dot as a fixation point was presented only under the fixation condition.

The standard stimulus comprised a moving dot and zero, one, two, four, six, or eight concentric circles (see Figure 7). A square rotated by 45° was used for the motion path of the dot. Under the conditions of two, four, six, and eight circles, the diameter of the outermost circle was a constant 10.0° and the diameter of the innermost circle was 5.0°, 2.5°, 1.7°, and 1.3°, respectively. The diameters of the other circles were set such that the circles were evenly spaced. Under the zero-circle condition, only a moving dot was presented. The number of intersections between the circles and the motion path of the dot was eight per revolution when there were four circles, eight per revolution when there were six circles, and 16 per revolution when there were eight circles. The moving dot and circles did not intersect when there were one or two circles. The dot motion path was never tangent to a circle under any condition.

Under the fixation condition, a black dot with a diameter of 0.14° was presented at the center of the standard stimulus as a fixation point (see Figure 7). No fixation dot was presented under the pursuit condition.

The other stimulus conditions were the same as those in Experiment 1.

#### Procedures

The task of the participants was to observe the motion trajectory of the dot while tracking the moving dot with the eyes (pursuit condition) or while staring at the fixation dot at the center of the standard stimulus (fixation condition), and to adjust the comparison stimulus to the same shape as the perceived motion path of the dot. At the start of a trial, an instruction was given on the screen as to which viewing style the participants should adopt.

There were a total of 96 trials for the six conditions of the number of circles and the two viewing conditions with eight repetitions.

Other procedures were the same as those in Experiment 1.

### Results and Discussion

**Figure 8.**
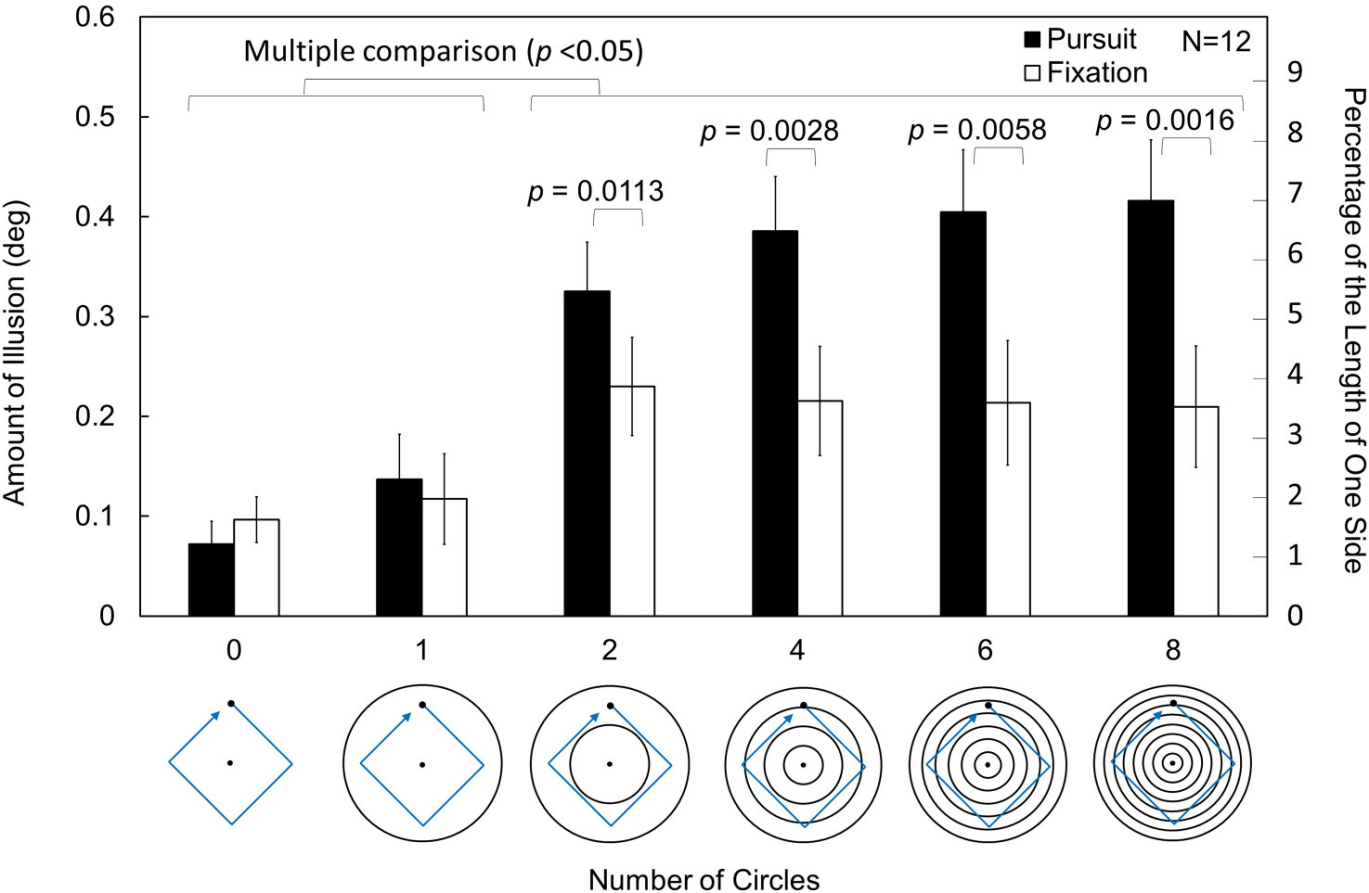
Results of Experiment 3. Under both viewing conditions, two or more circles produced a larger illusion. The large contribution of pursuit eye movement is evident for two or more circles. The *p* values indicate significant differences in the simple main effects. The results of multiple comparison tests (with an alpha level of 0.05) are also shown. There was no fixation dot under the pursuit conditions. The error bars indicate standard errors of means (SEs).

The amounts of the kinetic Orbison illusion under the 12 conditions are shown in Figure 8. A two-way repeated measures ANOVA was conducted. As Mauchly's test indicated the violation of sphericity, Greenhouse–Geisser's epsilon was used to adjust the degree of freedom. The main effect of the number of circles was significant, *F*(2.96,32.59) = 29.8159, *p *< .0001, *η_p_*²* *= 0.7305. The main effect of the viewing conditions was also significant, *F*(1,11) = 10.1002, *p *= .0088, *η_p_*²* *= 0.4787. A significant interaction was found between them, *F*(5,55) = 17.5578, *p *< .0001, *η_p_*²* *= 0.6148.

The simple main effects of viewing conditions were significant under the conditions of two, four, six, and eight circles, *F*(1,11) = 9.2118, *p *= .0113, *η_p_*²* *= 0.4558; *F*(1,11) = 14.6487, *p *= .0028, *η_p_*²* *= 0.5711; *F*(1,11) = 11.6328, *p *= . 0058, *η_p_*²* *= 0.5140; *F*(1,11) = 17.2336, *p *= .0016, *η_p_*²* *= 0.6104. No significant simple main effects of the two viewing conditions were found when there was no circle or one circle (*p* > .05).

The simple main effect of the number of circles under the pursuit condition was significant, *F*(2.7,29.67) = 34.6030, *p *< .0001, *η_p_*²* *= 0.7588. Multiple comparisons (made adopting the modified sequentially rejective Bonferroni method) revealed significant differences (*p *< .05) between the conditions of two, four, six, and eight circles and the conditions of no circle or one circle. No significant difference was found for the other pairs (*p *> .05). The simple main effect of the number of circles under the fixation condition was significant, *F*(2.92,32.07) = 10.3693, *p *= .0001, *η_p_*²* *= 0.4852. Multiple comparisons revealed significant differences (*p *< .05) between the conditions of two, four, six, and eight circles and the conditions of no circle or one circle. No significant difference in the illusion was found for the other pairs (*p *> .05).

The illusion was larger for two, four, six, and eight circles than for no circle or one circle, and the number of concentric circles thus affected the kinetic Orbison illusion. However, there was no significant difference between the conditions with multiple circles. This result indicates that the intersection of the motion path and circles is not a major factor in the kinetic Orbison illusion. When there were two circles, there was no intersection between the motion path and circles (i.e., the dot did not cross the circles). When there were eight circles, the motion path intersected with the circles 16 times in a cycle, yet there was no significant difference in the amount of illusion between the two-circle and eight-circle conditions as noted above.

The illusion was larger for pursuit eye movements than for eye fixation. Under the eight-circle condition, the amount of the illusion for pursuit eye movements was ∼ 1.98 that for staring at the fixation point. Thus, motion streaks are not an important factor in producing the kinetic Orbison illusion with multiple circles (two, four, six, or eight circles). This result is in clear contrast with that of Khuu (2012) showing the importance of motion streaks to the kinetic Zöllner illusion.

The fact that there is no difference in the amount of illusion between the conditions of no circle and one circle suggests that the contribution of the outer circle to this illusion is not large in the stimulus display in Experiment 3. The difference between the outer and inner circles might be another factor that affects the kinetic Orbison illusion, as investigated in Experiment 4.

Additionally, the fact that there is no difference between the fixation and pursuit conditions for no circle and one circle suggests that the measured illusion for no circle was qualitatively different from the illusion under the other conditions and that the illusion might be too weak to discriminate the two viewing conditions under the conditions of no circle and one circle.

### Additional Experiment

An additional experiment was conducted to examine the effect of viewing conditions during the adjustment of the comparison stimulus. The effect of eye movements during standard stimulus observation became clear, and it was thus necessary to examine the effect of eye movements during the adjustment of the comparison stimulus. To investigate whether the adjustment with free eye movement and that with gazing at the fixation point affected the measured amount of the kinetic Orbison illusion, we examined the effects of combinations of the two factors, namely the combination of fixation and pursuit conditions during observation of the standard stimulus and the combination of fixation and free eye movement conditions during adjustment.

There were 11 participants (age: mean of 24.18 years, SD of 2.98 years, nine females and two males). The standard stimuli were the same as for the eight-circle condition in Experiment 3, which generated the strongest illusion. As in Experiment 2, a black dot was placed at the center of the standard stimulus under the fixation condition. No fixation dot was presented under the pursuit condition. The comparison stimuli were the same as for the 45° orientation condition in Experiment 2. When the viewing condition during adjustment was a fixation, a fixation dot having the same size and color as the standard stimulus was presented at the center of the comparison stimulus; when the viewing condition during adjustment was a free observation, there was no fixation dot in the comparison stimulus.

**Figure 9.**
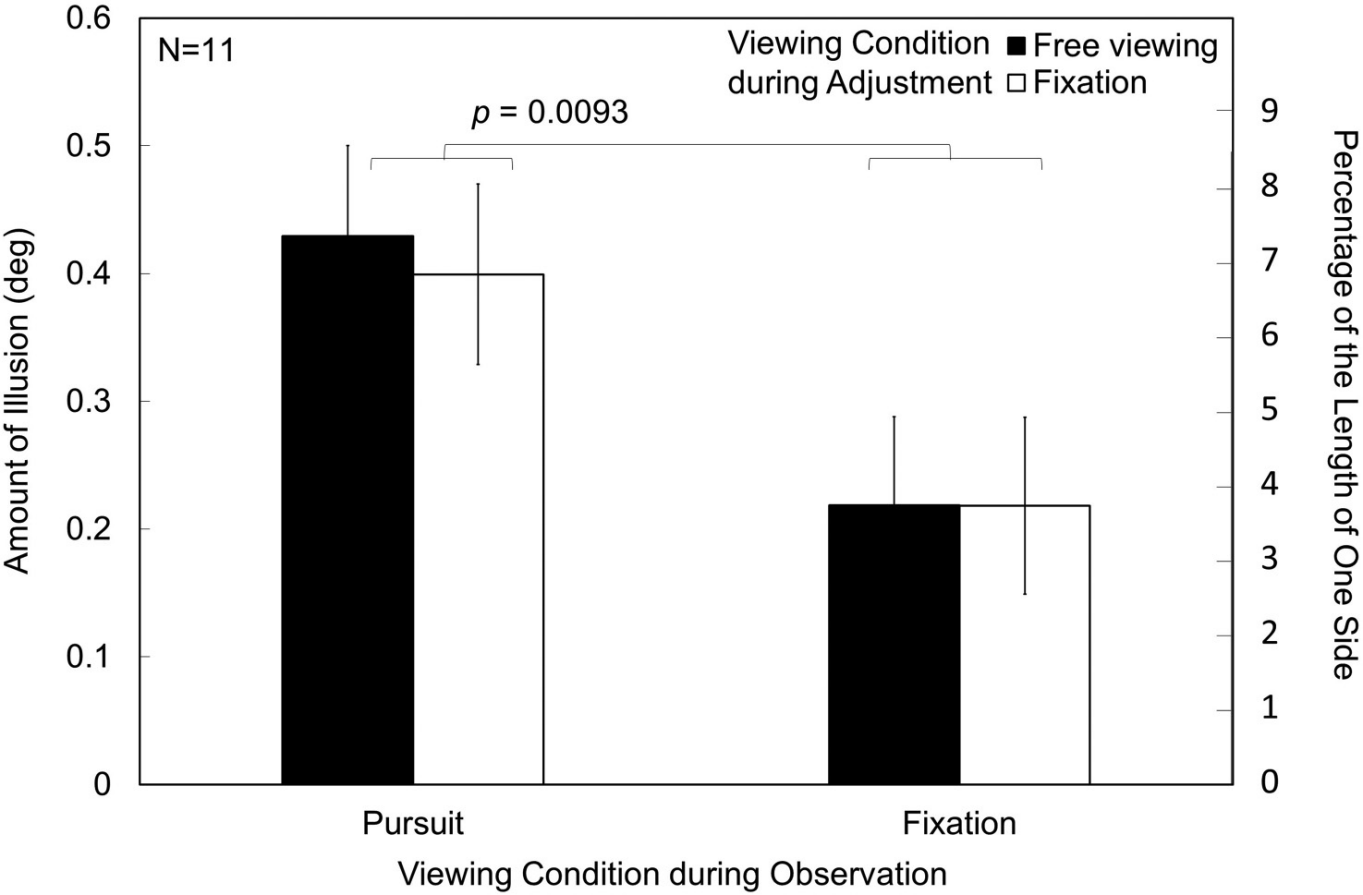
Results of the additional experiment. There was no effect of viewing conditions during the adjustment of the comparison stimuli. The *p* value indicates the significant difference in the main effect of the viewing condition during standard-stimulus observation. The error bars indicate standard errors of means (SEs).

The results are shown in Figure 9. The maximum amount of illusion was 0.43° (7.3% of the side length) under the pursuit condition. This value is the largest among the results in the present article. In a two-way repeated measures ANOVA, the main effect of the viewing conditions during standard stimulus observation was significant, *F*(1,10) = 10.3351, *p *= .0093, *η_p_*²* *= 0.5082, as in the previous experiment. The results confirm the reproducibility of the results for the viewing condition during standard-stimulus observation. However, the main effect of viewing conditions during adjustment was not significant, *F*(1,10) = 2.2430, *p *= .1651, *η_p_*²* *= 0.1832. The results indicate that the eye movement during adjustment when observing the comparison stimulus does not affect the results of adjustment. The measured amount of illusion thus reflects the illusion from the standard stimulus observation irrespective of the viewing condition during adjustment.

## Experiment 4

Experiment 4 was conducted to examine the effects of the inner and outer circles on the kinetic Orbison illusion. The outer circle was used for the one-circle condition in Experiment 3. We compared the effects of the inner and outer circles on the illusion.

### Method

#### Participants

There were 13 participants (age: mean of 24.46 years, SD of 3.08 years, 13 females).

#### Apparatus

The experimental setup was the same as that in Experiment 1.

#### Stimuli

The standard stimulus comprised a moving dot and circle(s). There were four circle conditions, namely the conditions of no circle, the inner circle only, the outer circle only, and both circles. The diameter of the outer circle was 10.0° and that of the inner circle was 3.6°. The distance at which the dot and outer circle were closest to each other (at the corners of the square path) and the distance at which the dot and inner circle were closest to each other (at the midway points of the square sides) were the same; that is, 1.13°.

#### Procedure

There were a total of 64 trials per participant for two conditions of the inner circle (with and without the inner circle), two conditions of the outer circle (with and without the outer circle), two viewing conditions (pursuing the moving dot and gazing at a fixation dot), and eight repetitions. The trials were conducted in random order.

Other procedures were the same as those in Experiment 3.

### Results and Discussion

**Figure 10.**
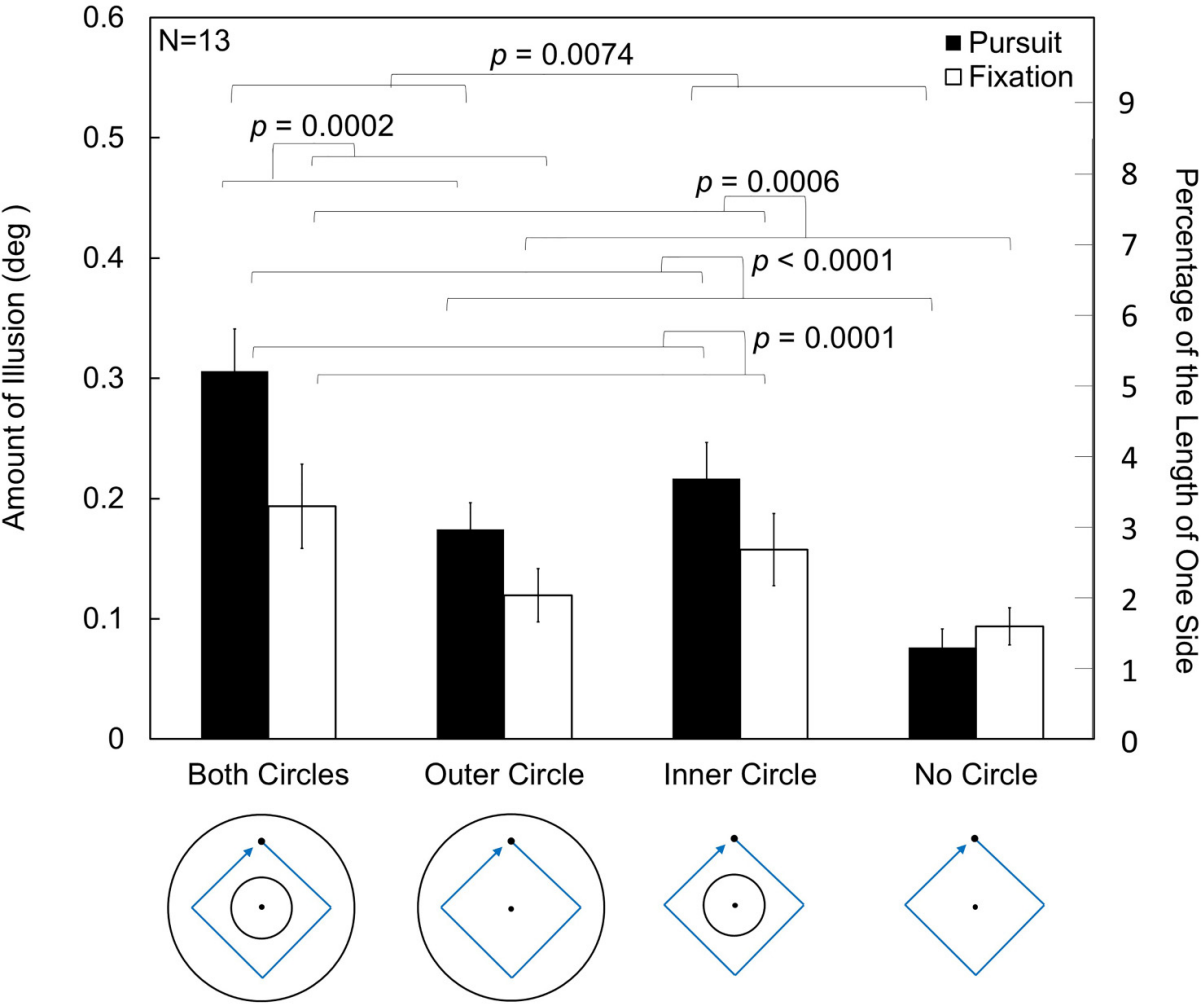
Results of Experiment 4. The presence of the inner or outer circle contributed to the illusion. There was no fixation dot under the pursuit conditions. The *p* values indicate the significant differences in the simple main effects. Error bars indicate standard errors of means (SEs). See details in the text.

The results of Experiment 4 are shown in Figure 10. A three-way repeated measures ANOVA was conducted. Main effects were found for the viewing conditions, presence of the inner circle, and presence of the outer circle, *F*(1,12) = 16.0426, *p *= .0017, *η_p_*²* *= 0.5721; *F*(1,12) = 42.2084, *p *< .0001, *η_p_*²* *= 0.7786; *F*(1,12) = 6.7064, *p *= .0237, *η_p_*²* *= 0.3585.

There was a significant interaction between the viewing conditions and the presence of the inner circle, *F*(1,12) = 24.4327, *p *= .0003, *η_p_*²* *= 0.6706. The simple main effect of the viewing conditions was significant under the condition with the inner circle, *F*(1,12) = 30.1794, *p *= .0001, *η_p_*²* *= 0 .7155, but not significant under the condition without the inner circle, *F*(1,12) = 3.0288, *p *= .1074, *η_p_*²* *= 0.2015. The simple main effect of the presence of the inner circle was significant under both pursuit and fixation conditions, *F*(1,12) = 52.6562, *p *< .0001, *η_p_*²* *= 0.8144; *F*(1,12) = 21.5459, *p *= .0006, *η_p_*²* *= 0.6423.

There was a significant interaction between the viewing conditions and the presence of the outer circle, *F*(1,12) = 14.5001, *p *= .0025, *η_p_*²* *= 0.5472. The simple main effect of the viewing conditions was significant under the condition with the outer circle, *F*(1,12) = 28.9929, *p *= .0002, *η_p_*²* *= 0.7073, but not under the condition without the outer circle, *F*(1,12) = 2.5506, *p *= .1362, *η_p_*²* *= 0.1753. The simple main effect of the presence of the outer circle was significant under the pursuit condition, *F*(1,12) = 10.3581, *p *= .0074, *η_p_*²* *= 0.4633, but not under the fixation condition, *F*(1,12) = 1.9591, *p *= .1869, *η_p_*² = 0.1403.

In this experiment, the main effects of both the inner and outer circles being present were observed. The fact that there was no interaction between them suggests that the effects of the outer and inner circles are independent of each other and that the effects could be summed when both circles exist. In a comparison between inner and outer circles, the effect size of the presence of the inner circle (*η_p_*²* *= 0.7786) was higher than that of the outer circle (*η_p_*²* *= 0.3585), suggesting that the inner circle had a greater effect than the outer circle in generating the kinetic Orbison illusion. However, a posthoc analysis adopting two-way repeated measures ANOVA to compare the outer circle and inner circle conditions showed that the difference was not significant (*p *> .05). Two points should be considered in further investigating the difference between inner and outer circles. The first is that the average distance between the dot and circle was larger for the outer circle in Experiment 4 although the closest distance was the same for the inner and outer circles. The other is that the curvature was larger for the inner circle because the radius was smaller. As these factors might affect the illusion, a more controlled experiment is required for clarification.

The effect of the viewing conditions was observed again, similar to the results obtained in Experiment 3. The contribution of the pursuit eye movement to the illusion seems to be clear and stable. The interactions seen in the statistics may arise mainly from there being no effect of the viewing conditions under the no-circle condition as shown in Experiment 3. The illusory effect measured under the no-circle condition was observed also in Experiment 2, though the measured illusion was not large. This effect might have an origin different from that of the illusion that we investigate here as noted before and as discussed later.

## Experiment 5

Experiment 5 was conducted to examine the effect of the speed of the moving dot on the kinetic Orbison illusion under the two viewing conditions. Under the pursuit eye movement condition, as the speed increases, pursuing the dot would become more difficult. If a smoother pursuit produces a larger illusion, the lowest speed would be the best condition for generating the illusion. In contrast, under the fixation condition, as the speed increases, motion streaks would become longer. If the motion streaks are important for the illusion (Khuu, 2012), the highest speed would be the best condition for generating the illusion.

### Method

#### Participants

There were 13 participants (age: mean of 24.46 years, SD of 3.08 years, 13 females).

#### Apparatus

The experimental setup was the same as that in Experiment 1.

#### Stimuli

The standard stimulus comprised eight concentric circles and a moving dot. The concentric circles were the same as those under the eight-circle condition in Experiment 3 (see Figure 7). The speed of the moving dots was set at four levels: 1.74°/s (13.25 s/rev), 3.48°/s (6.87 s/rev), 8.7°/s (2.68 s/rev), and 17.4°/s (1.34 s/rev). The slowest moving dot was easy for participants to pursue (Supplemental Movie 2) whereas the fastest moving dot was difficult for them to smoothly pursue (Supplemental Movie 3), inducing saccades. Under the fastest speed condition (17.4°/s), it took only 0.33 s for the dot to travel one side of the square path. The dot position was refreshed at a rate of 60 times per second.

#### Procedures

There were a total of 64 trials per participant for the four dot-speed conditions, two observation conditions (pursuit and fixation), and eight repetitions.

Other procedures were the same as those in Experiment 1.

### Results and Discussion

**Figure 11.**
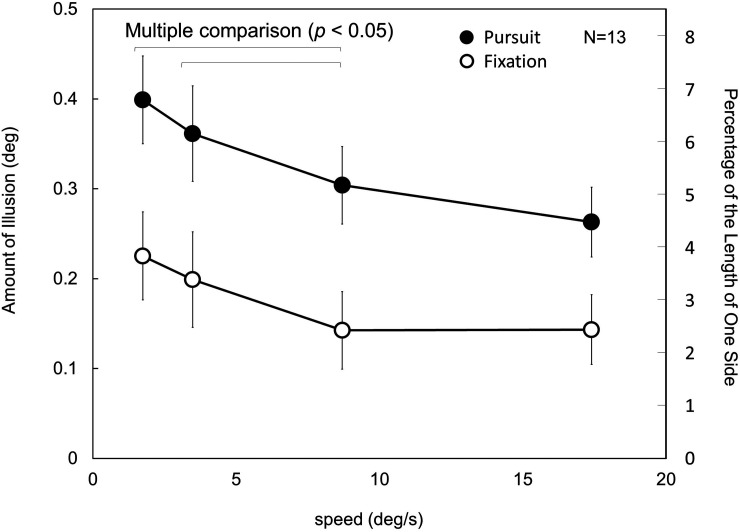
Results of Experiment 5. Under both viewing conditions, the lowest dot speed resulted in the strongest illusion. The results of multiple comparison tests are also presented. See details in the text.

The results of Experiment 5 are shown in Figure 11. A two-way repeated measures ANOVA was conducted. As Mauchly's test indicated the violation of sphericity, Greenhouse–Geisser's epsilon was used to adjust the degree of freedom. There were significant main effects for both the eye movement and dot speed, *F*(1,12) = 45.4253, *p *< .0001, *η_p_*²* *= 0.7910; *F*(1.34,16.13) = 4.8643, *p *= .0333, *η_p_*²* *= 0.2884). No interaction between them was found, *F*(1.97,23.58) = 1.2113, *p *= .3150, *η_p_*²* *= 0.0917. Multiple comparison tests (conducted adopting the modified sequentially rejective Bonferroni method) showed that there were significant differences between the dot-speed conditions of 1.74 and 8.7°/s and between the dot-speed conditions of 3.48 and 8.7°/s (*p *< .05).

The results demonstrate the effects of the pursuit eye movement and the dot speed. The effect of the pursuit eye movement has been repeatedly described in this article and was again found under all dot-speed conditions in Experiment 5. A dot moving at a lower speed may be easier for participants to pursue. The results show the tendency that a lower speed produced a larger illusion. This tendency appears to be consistent with the result of Cesàro and Agostini (1998) that the slalom effect decreased with an increase in the dot speed from 0.78 to 3.11°/s. However, other literature has reported a different tendency. Swanston (1984) reported that there was no difference in the kinetic Zöllner illusion between dot speeds of 2 and 4°/s. Khuu (2012) reported that the kinetic Zöllner illusion increased with an increase in the dot speed from 0.5 to 16°/s. As Swanston (1984) and Cesàro and Agostini (1998) seem not to have used a fixation point, discussion on the effect of pursuit eye movement/fixation may not be possible. However, the advantage of the lower speed was also seen under the fixation condition in Experiment 5. Under the fixation condition, a faster dot motion may produce longer motion streaks. However, even under the fixation condition, the lower speed is favored by the illusion. This point is difficult to explain from the view of motion streaks.

Additionally, even under the fastest speed condition, the illusion was greater for the pursuit condition. This suggests not that the smooth pursuit eye movement itself contributes to the illusion because precise pursuit may have been difficult owing to the excessive speed of dot motion in revolution. Instead, it suggests that the motion streaks hindered the kinetic Orbison illusion. The fixation condition and the faster speed conditions would be better conditions under which to produce motion streaks. The pursuit condition and the slower speed conditions would shorten or disperse motion streaks. Thus, the results in Experiment 5 suggest that the kinetic Orbison illusion becomes strong under conditions that there are no motion streaks or the motion streaks are shorter. However, it is noteworthy that there are individual differences. Three of the 13 participants showed more or less opposite tendencies in that a higher speed produced a larger illusion.

## General Discussion

### Summary

The purpose of this study was to qualitatively and quantitatively describe the kinetic Orbison illusion and to investigate the factors that cause the illusion. In Experiment 1, we demonstrated that the kinetic Orbison illusion and the Orbison illusion with a stationary figure had similar illusory tendencies and that the amount of illusion was larger for the kinetic Orbison illusion. In Experiment 2, we showed that the illusion was effective when the background circles were present and when the square motion path was tilted 45°. In Experiment 3, we examined the effects of the number of background circles and the viewing conditions. The results show that multiple circles (two to eight circles) produced a larger illusion than no circle or one circle. It was also shown that the intersections of the motion path and the circles or the crossing of the background circles were not important for the illusion to occur. Furthermore, the pursuit condition produced a larger illusion than the fixation condition. In Experiment 4, we showed that each of the outer and inner circles contributed to the illusion and that the effects of the inner and outer circles could be summed when the circles were presented together. The results again show that the illusion was larger with pursuit eye movements than with fixation. In Experiment 5, we showed that the illusion was greater with pursuit eye movements than with fixation and that a lower speed of dot motion produced a larger illusion, statistically. We conclude from these results that the phenomenon of the kinetic Orbison illusion has been clearly demonstrated and that the characteristics of the kinetic Orbison illusion are different from those of other reported kinetic geometrical illusions.

### Role of Motion Streaks

We conclude that the contribution of motion streaks to the kinetic Orbison illusion is not large, relative to that of the kinetic Zöllner illusion reported by Khuu (2012). In our experiments, the amount of illusion was lower under conditions for which motion streaks are supposed to be produced. In Experiments 3, 4, and 5, the pursuit condition consistently produced greater effects than the fixation condition. In Experiment 5, the motion velocity of the dots varied between 1.74 and 17.4°/s, and a dot at a lower speed produced a larger illusion even under the fixation condition. Khuu (2012) conducted experiments with dots traveling at speeds from 0.5 to 16°/s, as noted above, and found that the illusory tilt of the dot motion direction increased with the speed. In the kinetic Orbison illusion, it is not the case that a higher speed that is supposed to produce longer motion streaks produces a larger illusion.

We guess that the motion streaks even weaken the kinetic Orbison illusion. According to Geisler’s (1999) model, motion streaks contribute to the precise determination of the direction of motion. It is natural that motion streaks weaken an illusion of motion direction. Under the pursuit eye movement condition, the motion direction of the moving dot is mainly determined from information on mechanisms relating to eye movement and not from information on mechanisms relating to retinal motion and motion streaks. Therefore, the determination of the motion direction may be less precise under the pursuit condition, resulting in a larger illusory effect.

However, it is also true that, in Experiments 3, 4, and 5, the participants experienced the kinetic Orbison illusion under the fixation condition. Under this condition, information on the motion direction from the retinal motion may suggest the true motion direction. However, when orientation-selective cells responding to motion streaks interact with orientation-selective cells responding to the background circles, the motion streaks may affect the determination of the motion direction (Khuu, 2012). Thus, under the fixation condition, there were two opposing factors promoting or preventing the kinetic Orbison illusion. The individual difference found in Experiment 5 might reflect the contradiction. Under the pursuit condition, there should be little effect of motion streaks. We then ask, what was the cause of the illusion under the pursuit condition? We propose a hypothesis in the final section.

### Role of Intersections

In Experiment 3, there was no significant difference in the amount of illusion between the two-circle and eight-circle conditions. Under the two-circle condition, the dot never crossed a circle whereas the dot crossed the concentric circles 16 times per revolution under the eight-circle condition. Therefore, the intersections of the dot-motion path and circles were not a primary factor in generating the kinetic Orbison illusion regardless of whether observers pursued the moving dot or fixated on a central dot.

This result also differs from Swanston’s (1984) and Cesàro and Agostini’s (1998) claim that the trajectory of a dot plays the same role as a line segment, as subjective contours, and that kinetic illusions are caused by the virtual intersections of the dot trajectory and the line segments just as in real geometrical illusions, such as the Hering and Zöllner illusions.

When the static Orbison illusion figure comprises two circles without intersections, the illusion hardly arises (see Figures 7 and 10). In contrast, the kinetic Orbison illusion almost fully arises even with two circles (see Figure 8). The motion path in the kinetic Orbison illusion is not simply the substitution of the real lines in the Orbison illusion but more than that. In fact, Experiment 1 showed that the amount of the kinetic Orbison illusion was 2.36 times that of the static Orbison illusion. We should consider a factor lying in motion perception as noted later.

### Central Versus Peripheral Vision

A potential reason for the strong kinetic Orbison illusion under the pursuit condition is that the moving dots and background circles are seen in the central (or near central) field of view. In central vision, because the spatial resolution is higher, the positional relationship between a dot and a background circle (and its change) is seen clearly. Under the fixation condition of the present study, the fixation point was located at the center of the circles, such that even the dot position closest to the fixation point when moving on the square path was 2.95° away. However, it is also possible that the lower resolution is favored by the illusion through spatial ambiguity. In future studies, it will be necessary to separate the effect of central vision from that of pursuit eye movements.

### Some Unknown Factor

In Experiment 2, the kinetic Orbison illusion occurred even when there were no background circles or intersections. This residual effect, though not large, was still measured even for an orientation of 0°. The effect cannot be explained by the overshoot of pursuit eye movement at the corners of the square motion path because the effect remained under the fixation condition.

One possible reason for the illusion without the background circles is the aftereffect of the curvature. Although the mask stimulus was presented on the full screen after the completion of the adjustment, we cannot rule out the possibility that adaptation to the background circles gradually accumulated during the experiment, particularly for long-time viewing observers.

Another possible reason is that an artifact biased our observers to give responses indicating bending toward the center of the square. The measurement method adopted in the experiments required participants to adjust the shape of the comparison stimulus by bending the sides inward; that is, bending the shape outward was not possible. This could shift the response baseline (indicating no illusion) toward the inward bending direction. Although our observers did not assert the need to bend the shape outward, we should not exclude a subconscious bias of bending the shape inward in the responses.

After completing the experiments, the three authors qualitatively observed the stimuli again for a square trajectory with a 0° orientation and no background. We observed a trajectory in which the right angles of corners were slightly acute when the center of the image was fixated. This appearance could explain the small amount of curvature in the responses. Thus, in addition to the response artifact and curvature aftereffect, there may be an additional effect that bends the perceptual trajectory although it would be small and unexplained. The three possibilities may not be exclusive to each other.

### A New Hypothesis

The kinetic Orbison illusion may be affected by multiple factors, such as the orientation of the square motion path, viewing conditions (pursuit vs. fixation), and speed of dot motion. Although the oblique orientation is an illusion-enhancing factor, possibly related to the square–diamond illusion or the oblique effect, it is not a main cause of the kinetic Orbison illusion. The viewing condition and dot speed may relate to the production of motion streaks, a possible cause of kinetic geometrical illusions. However, in our experiments, the illusion was small when motion streaks should have been long or salient.

We here propose a new possible mechanism for the kinetic Orbison illusion, based on a motion interaction between motions of the dot and circles. Under the pursuit condition, there is no motion streak for the moving dot. However, the circle image should move on the retina in a direction that opposes that of the dot motion. Even in a situation where there is no intersection of the dot motion trajectory and circles, an interaction between dot and circle motion perceptions may be possible.

**Figure 12.**
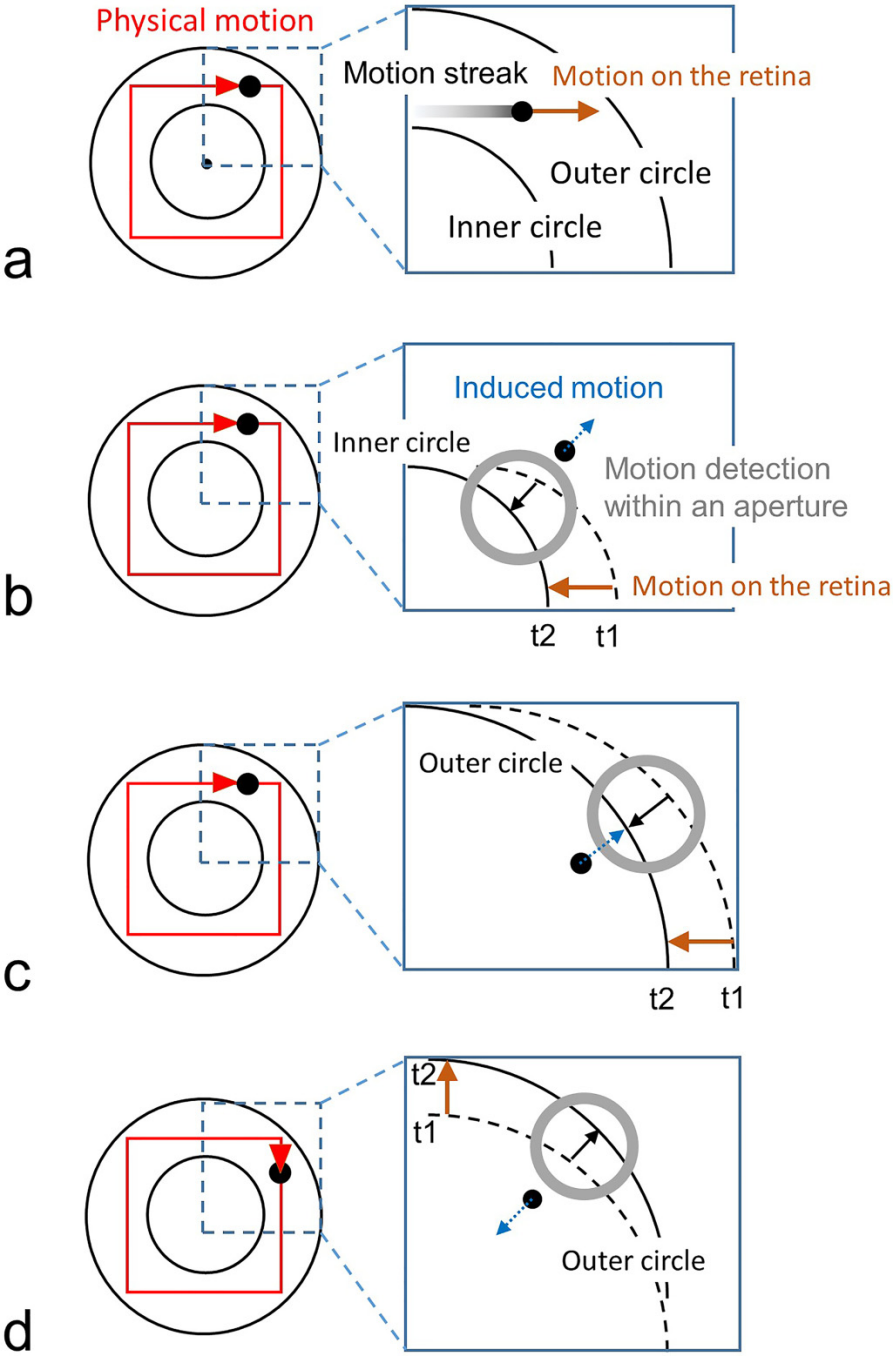
A new hypothesis for the kinetic Orbison illusion. (a) The motion of the image on the retina when the dot moving to the right at the upper right part of the square orbit of an upright square is observed with fixation. (b) Movement of the image of the inner circle on the retina when observed following the dot moving to the right in the upper right portion of the upright square orbit. The circular image at the *t*1 timing (broken circle) moves to the left and reaches the position of the *t*2 timing (solid circle). The inner circle moves to the left, but locally the detection of motion to the lower left is dominant owing to the aperture problem for motion. The dot motion may be induced to the upper right. (c) Movement of the image of the outer circle on the retina when observed following the dot moving to the right in the upper right portion of the square path. The outer circle moves to the left, but locally the detection of motion to the lower left is dominant owing to the aperture problem. The dot motion is induced to the upper right. (d) Movement of the image of the outer circle on the retina when observed following the dot moving down the upper right portion of the square path. The outer circle moves up, but the detection of motion to the upper right is locally dominant. The dot motion is induced to the lower left.

Figure 12 illustrates the proposed hypothesis. While the dot physically moves along an upright square path, the circle is stationary. When the dot is viewed with fixation, as shown in Figure 12(a), a motion streak occurs. The streak is a possible cause of the illusion while it is also a clue that suggests the correct direction of the dot motion. When a dot moving to the right in the upper right portion of the square path is pursued, motion to the lower left is locally detected for the inner circle owing to the aperture problem for motion (Wallach, 1935; Adelson & Movshon, 1982) as shown in Figure 12(b), and motion to the lower left is detected for the outer circle as shown in Figure 12(c). A relative motion component arises between the dot and the locally detected circle-edge motion. When this relative motion component generates induced motion of the dot, a motion component in the upper-right direction may be perceptually added to the rightward dot motion. Panel (d) illustrates the motion of the outer circle on the retina when the dot moves around the upper-right corner of the square's path in a clockwise direction and goes down. The outer circle moves up, but locally the motion is detected toward the upper right and is expected to generate induced motion of the dot to the lower left. According to this hypothesis, it is not necessary for the motion trajectory to intersect the background circle in creating the illusion. Both inner and outer circles would produce the illusion and the effects could be summed, which is consistent with the present results.

Swanston’s (1984) and Khuu’s (2012) claim was based on the interaction between virtual contours and real lines (i.e., a shape–shape interaction) causing errors in the motion direction. Our hypothesis claims that induced motion generated by the relative motion component between the dot motion and the local motion of circles (i.e., motion–motion interaction) causes errors in the perceived motion direction. This approach may be new for research on kinetic geometrical illusions.

Bressan and Vezzani (1995) and Ito et al. (2009) reported the illusory motion perception of physically static oblique lines due to the aperture problem for motion during the smooth pursuit of a moving dot. In traditional induced-motion experiments, whether a moving object is tracked by eye or not might hardly affect the amount of illusion (Brosgole et al., 1968; Schulman, 1979). However, we could not find studies on the motion induced by oblique retinal motion due to smooth pursuit eye movement. This point remains to be investigated.

It is important to note that we propose the addition of a possible mechanism for the illusion, and we do not assert that our hypothesis is the only mechanism of the kinetic Orbison illusion. This motion interaction effect could create the kinetic Orbison illusion only under the pursuit condition. This account is clearly not applicable to the appearance of the illusion under fixation conditions or that of the static Orbison illusion, although the structure of the illusory effect is qualitatively similar in all three cases. The present hypothesis should be tested in further experimental research.

## Supplemental Material

**Figure:** Video 1

**Figure:** Video 2

**Figure:** Video 3
